# Decorative Art

**Published:** 1895-02-09

**Authors:** 


### THE STUDY OF MAN.

### VIII.—DECORATIVE ART.

MAN has an instinctive appreciation of a quality in his surroundings which he calls Beauty, and which he has failed to analyse into anything else, and his instinct leads him to acquire and surround himself with beautiful things that his delight in them may be the more constantly gratified. Further, he finds it in his power to make things beautiful by his own handiwork. He has, that is, an artistic instinct, and becomes an artist to gratify it.

The artist works in one of two ways. Either he modifies his material, let us say a chance pebble, his club, his basket, or his face, so as to intensify those accidental qualities of form or colour which seem to him beautiful in it; or else he reproduces, in or on a material of one kind, the likeness of another object—a tree, a dog, or his brother man, which has formerly pleased him by its beauty, and which he desires to "have by him" in a more convenient form; at the same time, if he is wise, enhancing its essential qualities as before. In the latter case his art is representative; in the former it is merely decorative.

Representative art has been for centuries the theme of acute and elaborate theoretical criticism, and more recently of a historical criticism which has recovered many of its earlier phases, and revealed much of its esssential nature. But decorative art has been studied, if at all, from the craftman's point of view—successful "patterns" have been collected, and a working "theory of ornament" has grown up; but the subject has for the most part seemed beneath the dignity of serious or systematic research.[1]

The study, however, which has been concentrated upon the earlier stages of human civilisation of late years, could hardly fail to bring out the fact that throughout, and in the region of decorative no less than in that of representative art, progress is made from simpler to more complicated designs, and methods of execution; and that the modes of this progress seem to be referable to a number of apparently fundamental principles. It is in representative art, indeed, that this is the less clear; partly because, in the beginnings of art, direct representation occurs but rarely, and so the conditions under which it first makes its appearance are already somewhat complicated; partly because in all primarily representative art there is a direct reference at each reproduction, to the natural object whose outward form is to be simulated. The artist has, in the first place, to produce something like his model, and must refrain from the expression of his delight in mere combinations of outline and colour, and of his own mastery over his materials.

Decorative art, on the other hand, begins with the beginnings of productive industry. It rests closely upon the instinctive sense of the intrinsic delightfulness of certain forms and colours, quite apart from their representative associations, and upon the equally instinctive desire of the craftsman to turn out implements or furniture shapely and pleasant to the eye, as well as adequate to their primary uses. And it has no natural limits, except the capacity of the material and the utensil to bear ornament, and of the artist to devise and execute it. Consequently, we have the widest range of time, both absolute and relative, within which we may expect to find examples of decoration, namely, from the beginning of industry to the present day, and the freest field for the origination and development of types and forms.

The study of the principles which underlie decorative art is, as has been already indicated, of very recent growth, and has not been at all widely cultivated. In fact, the earliest treatise which shows any grasp of its meaning is that of George Harris on the "Theory of the Arts," published in 1869; and elaboration of the subject in detail has been hitherto represented by but a few names—in America by those of W. H. Holmes, Cushing, and Goodyear; in England most notably with that of General Pitt Rivers, whose vast anthropological collection, lately presented by him to the University of Oxford, was mainly brought together in illustration of it. Moreover, the material is so copious and so sub-divided, and the general principles of the theory can be so well formulated in relation to single series of data, that such essays as have appeared are mostly of a special and departmental kind, and are chiefly to be found in a number of learned and inaccessible journals. It is with the greater satisfaction, therefore, that we refer to a little book published hardly more than a year ago—"The Evolution of Decorative Art," by Henry Balfour, M.A., F.Z.S., in which these general tendencies are analysed, practically for the first time.

Mr. Balfour has been for some years Curator of the Pitt Rivers Museum in Oxford, and has consequently had unusual facilities for comparing and co-ordinating the data; and though he would probably be the last to contend that his essay is an exhaustive treatment even of the more fundamental questions, he has certainly contrived, in the small compass of some 130 pages, to give a conspectus of our knowledge of ornamentation, which is not only clear enough to serve as a beginner's introduction, but contains much that is suggestive, and not a little that is new to more advanced students. At the same time it forms an admirable commentary and guide to the Pitt Rivers Museum, from which the majority of its illustrations are derived.

Mr. Balfour divides the history of the beginnings of decoration into three principal stages. First of all, natural or accidental peculiarities, which may be even defects from an utilitarian point of view, are recognised as beautiful, and emphasised so as to become the *motif* of a decorative scheme: sometimes they are likenesses to other objects which are thus enhanced; and consequently we find representative art first as a handmaid to the decorator. Secondly, natural effects, or peculiarities of one material, are reproduced in another; copied, and thus in fact *represented*, into other surroundings, not once, in most cases, but repeatedly. Thirdly, successive copying leads to variations from the original model, partly accidental, but partly, and especially later, designed. New resemblances are seen in an old ornament, and emphasised in their turn; essentials are exaggerated; non-essentials omitted; and we are involved in a maze of conflicting or co-operating tendencies, till in later and composite patterns, *motif* and accessory, design and background, portrait and pattern, become inextricably confused before the inexperienced eye.

Three points emerge: The development of decorative art depends upon psychological laws, which do not fundamentally vary among different races of men, though they do not necessarily lead to at all similar results in detail; its success depends upon the fitness of the ornament for its place, and this upon its representation, however remotely, of *something* naturally to be found there; and nothing is so common or so defective that the eye of the artist cannot find the "beauty line" in it, and turn it to account.

